# LncRNA SNHG14 promotes inflammatory response induced by cerebral ischemia/reperfusion injury through regulating miR-136-5p /ROCK1

**DOI:** 10.1038/s41417-018-0067-5

**Published:** 2018-12-14

**Authors:** Yu Zhong, Chao Yu, Wenyi Qin

**Affiliations:** 1 Department of Integrated Traditional Chinese and Western Medicine, The First Affiliated Hospital of Chongqing Medical University, Chongqing, China; 2 Department of Pneumology, Chongqing JiangBei Hospital of Traditional Chinese Medicine, Chongqing, China

**Keywords:** RNAi, Molecular biology

## Abstract

Recently, long non-coding RNAs (lncRNAs) are considered as critical regulators in pathogenesis progression of cerebral ischemia. In present study, lncRNA-small nucleolar RNA host gene 14 (SNHG14) was found upregulated in middle cerebral artery occlusion/reperfusion (MCAO/R) treated brain tissues and oxygen-glucose deprivation and reoxygenation (OGD/R) treated PC-12 cells. Interference of SNHG14 by shRNA vector enhanced neuron survival and suppressed inflammation in response to OGD/R insult. SNHG14 positively regulated the expression of Rho-associated coiled-coil-containing protein kinase 1 (ROCK1) via acting as a sponge of microRNA (miR)-136–5p. SNHG14 promoted neurological impairment and inflammatory response through elevating the expression of ROCK1 while decreasing miR-136–5p level in OGD/R induced damage. Collectively, we illustrated that SNHG14 could be a novel strategy for treatment ischemia stoke.

## Introduction

Stroke is considered as one of the most common causes of mortality and disability throughout the world [1]. It is characterized as an acute cerebrovascular disease and approximately 85% stroke cases are caused by cerebral ischemia, which occurs when embolic or thrombotic occlusion blocks the major cerebral artery [2]. Enormous efforts have been made to develop novel treatment strategies for stoke, however, up to date, thrombolysis by intravenous recombinant tissue plasminogen activator (tPA) is still the only clinically effective therapeutic treatment [3]. Still more, thrombolytic therapy is only benefit to a few stoke suffers due to its narrow treatment window [4]. Therefore, it is necessary and urgent to searching novel and effective therapeutic targets or prognosis biomarkers for stoke patients. Undoubtedly, a better understanding of the underlying molecular mechanism of cerebral ischemia will contribute to exploit effective neuroprotective strategies.

Recently, a growing number of studies reveal that long non-coding RNAs (lncRNAs) are tightly associated with various neurologic disorders including ischemia stoke [5]. LncRNAs are a family of non-functional protein coding RNAs including more than 200 nucleotides and participate in regulation a variety of biological processes [6]. Small nucleolar RNA host gene 14 (SNHG14), also known as antisense of ubiquitin protein ligase E3A (UBE3A-ATS), is a 19.2 kb transcript and located on chromosome 15q11.2, which hosts two small nucleolar. Due to overlapping the entire UBE3A gene and promoter in antisense direction, SNHG14 was initially found as a silencer of UBE3A expression [7], which deficiency in brain result in neurogenetic disorders [8, 9]. Thus, overexpression of SNHG14 and silencing of UBE3A expression were associated with neuronal differentiation in Angelman syndrome model [10]. Moreover, SNHG14 participated in promoting microglia activation by modulation miR-145–5p/PLA2G4A in cerebral infarction [9]. On the basis, several studies have suggested SNHG14 is a pivotal modulator in neuronal diseases, however, the role and potential mechanism of SNHG14 in the ischemia stroke still remains to be largely investigated.

Emerging evidences suggested that lncRNA possesses intrinsic miRNA sponging properties via acting as a competing endogenous RNAs (ceRNAs) [11]. miRNAs are short non-coding RNAs with approximately ~21 nucleotides and suppress mRNA translation by targeting its 3′ untranslated region (3′ UTR) [12]. In present study, bioinformation screened miR-136–5p have complementary base paired with SNHG14. MiR-136–5p could be a target of lncRNA in cuprizone-induced demyelination or glioma malignancy [13, 14]. Previous studies major focus on its roles in cancer or spinal cord injury [13, 14], however, the studies on the functional involvement of miR-136–5p and its potential regulatory mechanism in ischemic stroke are still rarely.

Rho-associated coiled-coil-containing protein kinase 1(ROCK1) is a Rho-binding protein with serine/threonine kinase activity [15]. ROCK1 extensively participates in modulation of ischemia/reperfusion induced injury in myocardial, live, kidney, or brain [16–18] and also acts as a “molecular switch” in inflammatory response in different inflammatory diseases [19]. In cerebral hypoxia-reoxygenation impairment, ROCK1/F-action worked as a promoter via enhancing mitochondrial apoptosis [20]. Taken together, our study demonstrated a novel lncRNA SNHG14/miR-136–5p/ROCK1 regulatory network in ischemia induce injury, providing a potential biomarker and therapeutic target for ischemia stroke.

## Materials and methods

### Middle cerebral artery occlusion (MCAO) and animal treatment

Adult male Sprague–Dawley rats (SD, 8–10 weeks of age and ~250 g in weight) were obtained from Shanghai Laboratory Animal Center (Shanghai, China). All animal experiments and procedures were performed in accordance with the guidelines in the First Affiliated Hospital of Chongqing Medical university Animal Center. Institutional Animal Use Policy and approved by the Institutional Animal Care and Use committee of the First Affiliated Hospital of Chongqing Medical university (No.CSTC,2008CA5003) . The animals were housed in cages maintained in a regulated environment of humidity and temperature (12 h light/dark cycle) with standard rat diet and water.

For focal cerebral ischemia, right middle cerebral artery occlusion (MCAO) were performed in SD rats. In brief, rats were anesthetized with isoflurane (3% initially, 1–1.5% maintenance) in N_2_O and O_2_ (3:1) and the rectal temperature was maintained at 37 ± 0.5 °C. After middle cervical skin was incised, common carotid artery (CCA), internal carotid artery (ICA) and external carotid artery (ECA) were carefully isolated. The middle cerebral artery (MCA) was occluded by a heparin-dampened nylon suture, which was inserted from CCA into ICA and then forwarded to the origin of MCA. Consequently, the blood flow to the right MCA was blocked. After occlusion for 1 h, the filament was slowly removed for reperfusion. Sham operated rats were only separated the right MCA without suture insertion. After reperfusion for 12, 24, and 48 h, the rats were deeply anesthetized and then brains were removed immediately for further study.

### In vivo SNHG14 knockdown

The lentiviral vectors containing shRNA SNHG14 or non-targeting control (sh control) were purchased from Shanghai Genechem Co (Shang, China). Lentiviral was injected into lateral ventricles using a stereotaxic instrument under isoflurane anesthesia. Stereotaxic coordinates used were: anteroposterior (AP) = 0.8 mm, mediolateral (ML) = ± 1.4 mm and dorsoventral (DV) = 3.5 mm relative to bregma. Lentiviral particles containing SNHG14 shRNA (1 × 10^8^ TU/mL, Dharmacon) or sh control were slowly injected over 10 min into each of the lateral ventricles (5 µL/hemisphere at a rate of 0.5 µL/min). Rats were then feeding for 14 days to allow for recovery, shRNA expression and subsequently SNHG14 knockdown. After verifying the knockdown efficiency on the first set of animals, the left animals were subjected to MCAO and for the further study.

### Infarct volume measurement

The infarct volume of the brain was evaluated using 2% 2,3,5-triphenyltetrazolium chloride (TTC) staining after reperfusion or sham operation. After anaesthetized, the brains were rapidly removed and froze in a −20 °C refrigerator for 30 min and then sliced into serial coronal sections (2 mm thick). The sections were incubated in 2% TTC (PH7.4, sigma, St Louis, MO) dissolving in phosphate-buffered solution (PBS) for 30 min at 37 °C. TTC-stained sections were photographed by Olympus microscope (Olympus, Tokyo, Japan). The infarcted area identified as unstained by TTC were measured by Image J software (National Institutes of Health, Bethesda, USA). The percentage of infarct size per slice was calculated as follows: (infarcted area /total brain area) × 100%.

### Cell culture and oxygen-glucose deprivation (OGD)

Rat adrenal pheochromocytoma cell line (PC-12) was acquired from Shanghai Institutes of Cell Biological Sciences (Shanghai, China). The cells were cultured in Dulbecco’s modified Eagle’s medium (DMEM, high glucose, Sigma) supplemented with 10% fetal bovine serum (FBS, Gibco, Grand Island, NY) and 100 U/mL penicillin/streptomycin (Gibco). The cells were maintained in a humidified incubator with 5% CO_2_ at 37 °C.

OGD treatment with PC-12 cells was performed when the cell density was appropriate. Briefly, PC-12 cells were transferred to a hypoxic chamber with 5% CO_2_, 95% N_2_ and cultured in serum/glucose-free DMEM medium at 37 °C for 3 h. After OGD exposure, the cells were maintained in the glucose-containing DMEM with 10% FBS under normoxic conditions for 12, 24, and 48 h to reoxygenation. Cells cultured under normal conditions were used as a control.

### Cell transfection

MiR-136–5p mimic for overexpression the miR-136–5p level and mimic control (NC mimics), small interfering RNAs (siRNA) for SNHG14 (si-SNHG14) or si-NC, pcDNA3.1- SNHG14 overexpression vector (pc-SNHG14) or its control pcDNA vector (pc-DNA), as well as SNHG14 knockdown plasmids (SNHG14 shRNA1, SNHG14 shRNA2, and SNHG14 shRNA3) or its negative control (Scr shRNA) were synthesized by GenePharma (Shanghai, China). After seeded at appropriated density, cells were transfected using Lipofectamine 2000 Reagent (Invitrogen, Carlsbad, USA) according to the manufacturer’s protocol. Cells were harvested at 24 h after transfection for the next study. The target sequences of SNHG14 shRNA1, SNHG14 shRNA2, and SNHG14 shRNA3 were listed in Table 1.

**Table 1:** List of SNHG14 shRNA sequences and primer sequences for qRT-PCR were used in the study

Name	Primer	Sequence (5′-3′)
shRNA1	Sense	5′-GCAAAUGAAAGCUACCAAU-3′
	Anti-sense	5′-AUUGGUAGCUUUCAUUUGC-3′
shRNA2	Sense	5′-GCACAAUAUCUUUGAACUA-3′
	Anti-sense	5′-UAGUUCAAAGAUAUUGUGC-3′
shRNA3	Sense	5′-CUAGAAUCCUAAAGGCAAA-3′
	Anti-sense	5′-UUUGCCUUUAGGAUUCUAG-3′
SNHG14	Forward	5′-GGGTGTTTACGTAGACCAGAACC-3′
	Reverse	5′-CTTCCAAAAGCCTTCTGCCTTAG-3′
GAPDH	Forward	5′-AATTCCATGGCACCGTCAAG-3′
	Reverse	5′-TGGACTCCACGACGTACTC-3′
IL-6	Forward	5′-AGTGAGGAACAAGCCAGAGC-3′
	Reverse	5′-AGCTGCGCAGAATGAGATGA -3′
IL-1β	Forward	5′-CAGAAGTACCTGAGCTCGCC-3′
	Reverse	5′-AGATTCGTAGCTGGATGCCG-3′
TNF-α	Forward	5′-GCTGCACTTTGGAGTGATCG-3′
	Reverse	5′-CTTGTCACTCGGGGTTCGAG-3′
U6	Forward	5′-CTCGCTTCGGCAGCACA-3′
	Reverse	5′-AACGCTTCACGAATTTGCG-3′
miR-136-5p	RT primer	5′-CTCAACTGGTGTCGTGGAGTCGGCAAT TCAGTTGAGTCCATCA-3′
	Forward	5′-ACACTCCAGCTGGGACTCCATTTGTTTT-3′
	Reverse	5′-CCAGTGCAGGGTCCGAGGT-3′

### MTT assay

MTT assay were used to evaluate survival of PC-12 cells with OGD treatment under knockdown of SNHG14 expression. PC-12 cells (10^4^ cells/cm^2^) were seeded into 96-well plates. Next, 15 µL/well MTT solution (5 mg/mL, Sigma) was added into each well and incubated at 37 °C for 4 h. The supernatant was dissolved in 150 μL DMSO and the optical density of each well was determined by the absorbance at 570 nm using a micro-plate reader (Bio-Tek, Winooski, USA).

### Quantitative reverse transcription-polymerase chain reaction (qRT-PCR)

Total RNA samples from PC-12 cells or brain tissues were extracted using TRIzol Regent (Invitrogen) according to the manufacturer’s instruction. For cDNA synthesis, 2 μg of total RNA was reverse transcribed using the PrimeScriptTM RT reagent kit with gDNA Eraser (Perfect Real Time) (Takara, Otsu, Japan). qPCR was carried out using SYBR Green PCR Master Mix (Takara) on a Bio-Rad Real-Time PCR instrument (Bio-Rad, Hercules, USA). Expression levels of SNHG14, interleukin (IL)-6, IL-1β, and tumor necrosis factor (TNF)-α were normalized to the glyceraldehyde-3-phosphate dehydrogenase (GAPDH) and miR-136–5p was standardized to that of U6. Fold changes were calculated with the 2^-ΔΔCt^ method and every data was performed in triplicate. The primers used for RT-qPCR are listed in Table 1.

### Western blot analysis

Total proteins were extracted from cells or tissues using RIPA lysis buffer (Sigma) on ice. The concentration was determined by a BCA detecting kit (Beyotime Biotechnology, Nanjing, China) according to the manufacturer’s instructions. Protein samples were separated by 10% SDS-PAGE, transferred onto a PVDF membrane (Millipore, Billerica, USA) and blocked with 5% skimmed milk powder solution for 2 h at room temperature. Next, membranes were incubated with primary antibodies: anti-caspase-3 antibody (Abcam, Cambridge, UK; dilution rates of 1:500), anti-ROCK1 (Abcam, dilution rates of 1:2000), and anti-GAPDH antibody (Abcam, dilution rates of 1:2000) at 4 °C overnight, respectively. The target proteins were then incubated with secondary HRP anti-Rabbit antibodies (Abcam, dilution rates of 1:2000) for 1 h at room temperature and visualized using an enhanced chemiluminescence detection system (Pierce, Rockford, USA). The signals were captured and intensities of bands were quantified using Image Lab™ Software (Bio-Rad).

### Fluorescence in situ hybridization (FISH) and Immunofluorescence (IF) assay

FISH was performed on fresh brain tissue slices for SNHG14 expression level analysis after different reperfusion time. In brief, slices were treated with 0.5% Triton X-100 dissolved in PBS buffer, then incubated with equal amount of anti-SNHG14 biotin labeled-doligodeoxy-nucleotide probes (RiboBio, Guangzhou, China) dissolved in hybridization solution in humid chamber at 37 °C overnight. The second day, slices were washed in 4× sodium citrate buffer (SSC) with 0.1% Tween-20 for three times at 42 °C, followed washed in 2× SSC and 1× SSC one time, respectively. Next day, the secondary Cy3-conjugated anti-biotin antibody (Abcam, dilution rates of 1:2000) was incubated for 1 h at room temperature and then incubated with DAPI (Beyotime biotechnology, Nanjing, China) for 5 min. The stained images were captured using an Olympus fluorescence microscopy.

For immunofluorescence (IF) analysis, the brain tissues were collected following ischemia/reperfusion (I/R) and cut in coronal frozen slices (20 μm). After dehydration and antigen retrieval, sections were blocked with 5% bovine albumin for 30 min at room temperature and then incubated with primary anti-ROCK1 antibody (Abcam, dilution rates of 1:500) at 4 °C overnight. Next day, the secondary Cy3-conjugated anti-Rabbit antibody (Abcam, dilution rates of 1:1000) was incubated for 1 h at room temperature followed with incubation with DAPI. The stained images were captured using an Olympus fluorescence microscopy.

### Lactate dehydrogenase (LDH) assay

Cell membrane integrity was determined using a commercial lactate dehydrogenase (LDH) kit (Roche, Basel, Switzerland) according to the protocol company’s instructions. The decrease of cell membrane integrity (e.g., increase in LDH levels) indicates cytotoxicity. In brief, the LDH levels in cell culture medium were assessed after cells treated with isoflurane and/or mild hypothermia. The amount of product was measured spectrophotometrically at a wavelength of 490 nm (SLT, Spectra). The cell integrity index was calculated as followed: LDH levels in the supernatant/ the total LDH levels.

### ELISA assay

To identify the alteration of IL-6, IL-1β, or TNF-α release in PC-12 cells in response to different treatments, IL-6, IL-1β, or TNF-α content of the cell culture supernatant were centrifuged at 200×*g* for 10 min to remove cell debris and then detected using ELISA kit (Sigma) following the manufacturer’s instruction. Data were expressed as pg/mL or ng/mL.

### Dual Calcein-AM/PI staining

The Calcein-AM/PI staining double stain kit (Yeasen, Shang, China) was used for live/dead cell analysis according to manufacturer’s instruction. After centrifuged at 1000 rpm for 3 min, PC-12 cells were resuspended by assay buffer at a concentration of 1 × 10^5^–1 × 10^6^/mL. Next, cells were incubated with calcein AM solution for 15 min at 37 °C and then stained with propidium iodide (PI) for 15 min. The calcein-AM-stained or PI-stained cells were visualized by Olympus fluorescence microscopy and dead cells stained by PI were quantified by Image J software ((National Institutes of Health).

### Caspase-3 activity assay

Caspase-3 activities were determined by caspase-3 activity assay kit (Abcam). The proteins were extracted from PC-12 cells and then added into 96-well plates with reaction buffer and substrate. The mixtures were incubated for 4 h at 37 °C in the dark and then assayed using a spectrophotometer (Bio-Rad) at a wavelength of 450 nm.

### Dual-luciferase reporter gene assay

The fragments from SNHG14 containing the predicted binding site miR-136–5p (SNHG14-WT) or the corresponding mutant produced by mutating the miR-136–5p seed region binding site (SNHG14-MUT), as well as the fragments from ROCK1 3′UTR containing the predicted miR-136–5p binding site (ROCK1-WT) or the corresponding mutants created by mutating the miR-136–5p seed region binding site (ROCK1-MUT) were constructed by The Beijing Genomics Institute BGI (Beijing, China) and then inserted into a pmirGLO Dual-luciferase miRNA Target expression Vector (Promega, Madison, USA). PC-12 cells were cotransfected with SNHG14-WT or SNHG14-MUT, as well as ROCK1 3′UTR-WT or ROCK1 3′UTR-MUT, followed transfecting with miR-136–5p-mimic or NC-mimic using Lipofectamine 2000 (Invitrogen). The relative luciferase activity was evaluated by normalizing the firefly luminescence to the Renilla luminescence using the Dual-Luciferase Reporter Assay System (Promega) according to the manufacturer’s instructions at 48 h after transfection.

### RNA immunoprecipitation

The RIP assay was used to detect the binding relationship between endogenous SNHG14 and miR-136–5p in PC-12 cells. After lysed in a RIP buffer (Beyotime biotechnology), the whole cell lysate was incubated with magnetic beads (Biomart, Beijing, China) conjugated with rabbit anti-Argonaute2 (Ago2) antibody (Millipore), or negative control immunoglobulin G (IgG, Millipore) for 6 h at 4 °C. Next, the RNA was extracted by TRIzol regent after washed by ice-cold saline water (150 mmol/L NaCl). The purified RNA expression were analyzed by qRT-PCR.

### RNA pull-down assay

RNA pull-down assay were performed for detecting whether SNHG14 can be pulled down by miR-136–5p in PC12 cells. miR-136–5p, miR-136–5p-mutant (miR-326-Mut) with disrupt base pairing between SNHG14 and miR-135–5p or its negative control (NC) were biotin-labeled using Biotin RNA Labeling Mix (Roche) and T7/SP6 RNA polymerase (Roche). After 48 h of transfection, cells were lysed and then incubated with Dynabeads M-280 Streptavidin (Dynal AS, Norway) and biotinylated miRNAs for 30 min at room temperature on a rotator. Next, bounding RNAs were obtained from beads and the expression of SNHG14 were determined by qRT-PCR.

### Hematoxylin-eosin (HE) staining

After animals were anesthetized, the brains were obtained and fixed in 4% paraformaldehyde. The brains were cut into slices in 4 μm thick after embedded in paraffin. The slices containing hippocampus and cortex region were treated with hematoxylin and eosin (HE) solutions. The images were captured by a light microscope (Olympus) at 4 × 10 and 40 × 10 magnification.

### Neurological deficit scores assessment

Neurological deficit scores were determined at 24 h after MCAO according to the criteria of Longa 5 scores [21]. The neurological deficit was graded as follows: 0 score, non-neurological deficit; 1 score, extending contralateral forelimb impairment; 2 score, circling to left; 3 score, fall forward to left; 4 score, no spontaneous walking and unconsciousness. The performer was blind to the treatments.

### Statistical analysis

All the data were indicated as mean ± standard deviations (SD) of three independent experiments and surveyed using the Statistical Package for Social Sciences version 17.0 (SPSS 17.0; SPSS, Inc., Chicago, IL) and the Prism statistical software package (Version 5.0; GraphPad Software, Inc.). A single comparison between two groups was analyzed by Student’s *t*-test, and multiple group comparisons were surveyed with one-way analysis of variance (ANOVA). A value of *P* < 0.05 was considered to be statistically significant for the differences.

## Result

### SNHG14 was highly expressed upon I/R in vitro and in vivo

**Fig. 1:**
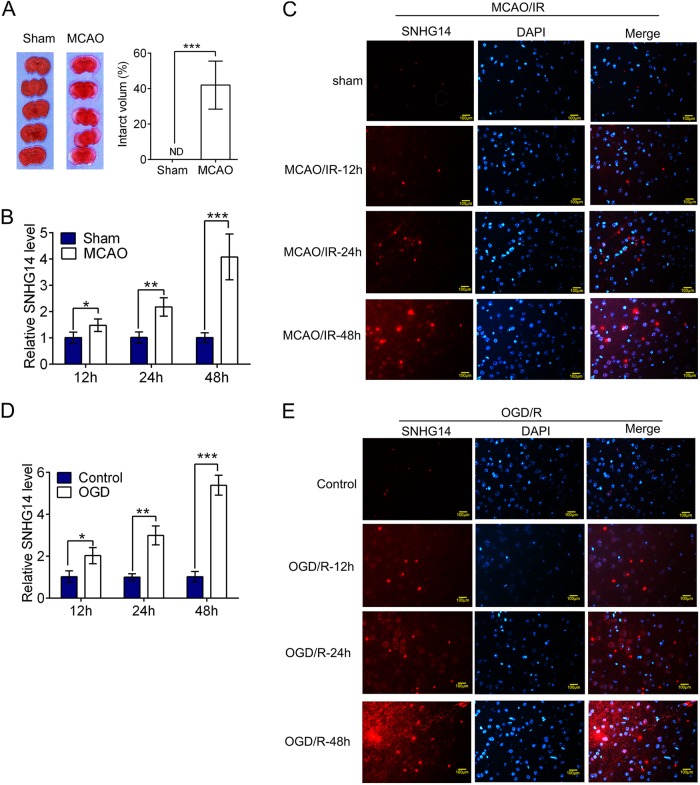
SNHG14 was upregulated following I/R in vitro and in vivo. **a** Representative brain sections of focal ischemia induced by MCAO were exhibited by TTC staining. Quantitative analysis of brain infarct volume after I/R in rats were shown by bar graph. **b** The expression of SNHG14 level was detected by qRT-PCR at 12, 24, and 48 h following MCAO/R in rats. **c** Representative images exhibiting the expression level and intracellular localization of SNHG14 were determined by RNA-FISH at 12, 24, and 48 h following MCAO/R in rats. **a**–**c** *N* = 6 per group. **d** The expression level of SNHG14 was examined by qRT-PCR in PC-12 cells after OGD/R. **e** Representative images exhibiting the expression level and intracellular localization of SNHG14 were determined by RNA-FISH in PC-12 cells after OGD/R. Data were expressed as mean ± SD. ***P < 0.001 represents statistically difference. Scale bar = 100 μm

To check the expression of SNHG14 in the pathophysiology of cerebral ischemia stoke, the MCAO operation induced reperfusion injury was verified by TTC staining. As shown in Fig. 1a, the infarct volume and brain damage were dramatically enhanced by MCAO operation compared with sham group (*P* < 0.001). Next, to evaluate the expression of SNHG14 in brain tissue following middle cerebral artery occlusion/ reperfusion (MCAO/R), focal cerebral ischemia in adult male rats was induced by MCAO for 1 h, followed by a reperfusion for 12, 24, or 48 h, then the levels of SNHG14 were determined by qRT-PCR and IF methods. The result showed that the expressions of SNHG14 were up-regulated after MCAO/R in a time dependent manner (12 h: *P* < 0.05, 24 h: *P* < 0.01; 48 h, *P* < 0.001, Fig. 1b), which also confirmed by the result of SNHG14 immunology staining (Fig. 1c), indicting its potential roles in ischemia. Further, PC-12 cells treated with oxygen-glucose deprivation and then reoxygenation for 12, 24, and 48 h were established for further measuring the expression of SNHG14 under ischemia condition in vitro. The expressions of SNHG14 were also enhanced in a time dependent way in oxygen-glucose deprivation/reoxygenation (OGD/R) treatment cells, which confirmed by qRT-PCR and IF assays (12 h: *P* < 0.05, 24 h: *P* < 0.01; 48 h, *P* < 0.001, Fig. 1d, e). The MCAO/R at 48 h and OGD/R at 48 h were chosen for the further experiments based on the above result. On the basis, SNHG14 expression were up-regulated in response to ischemia in MCAO/R induced rats and OGD/R treated cells, indicting its potential roles in ischemia stroke.

### Inhibition of SNHG14 decreased neuronal impairment and inflammation in response to OGD/R treatment in vitro

**Fig. 2:**
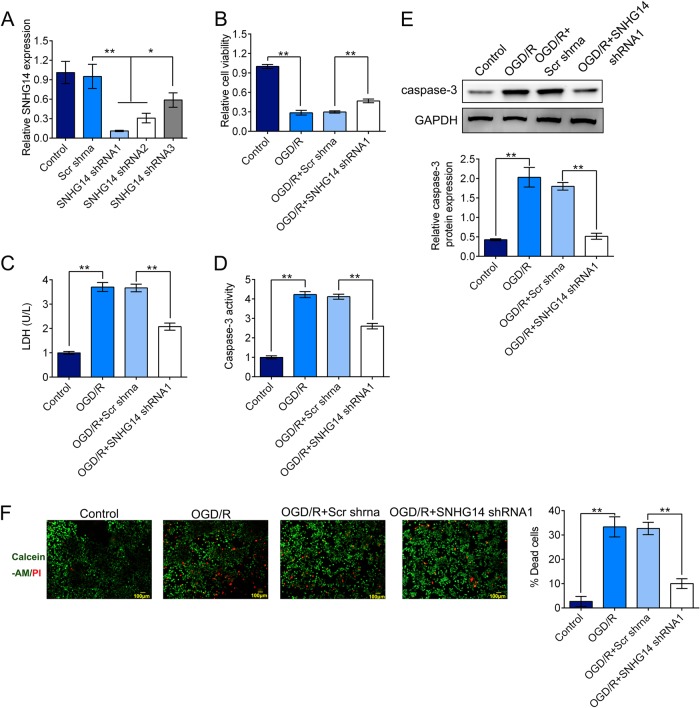
Silencing of SNHG14 inhibited cell injury induced by OGD/R in PC-12 cells. **a** The expression level of SNHG14 were measured by qRT-PCR in PC-12 cells transfected with shRNAs targeted SNHG14 (SNHG14 shRNA1, SNHG14 shRNA2, SNHG14 shRNA 3) or its control vector (Scr shRNA). **b** The effect of SNHG14 silencing on PC-12 cell viability was determined by MTT assay. **c** The role of knockdown of SNHG14 expression on the LDH concentration was measured by LDH releasing assay. **d**, **e** The effect of downregulated SNHG14 on caspase-3 activity and protein level were examined by caspase-3 activity assay and western blot in PC-12 cells, respectively. **f** The function of decreased SNHG14 on the number of dead cells was detected by dual calcein-AM/PI staining. The calcein-AM stained- (green) and PI- (red) stained cells represented live and dead cells, respectively. Data were expressed as mean ± SD. *P < 0.05, **P < 0.01 represents statistically difference. Scale bar = 100 μm

**Fig. 3:**
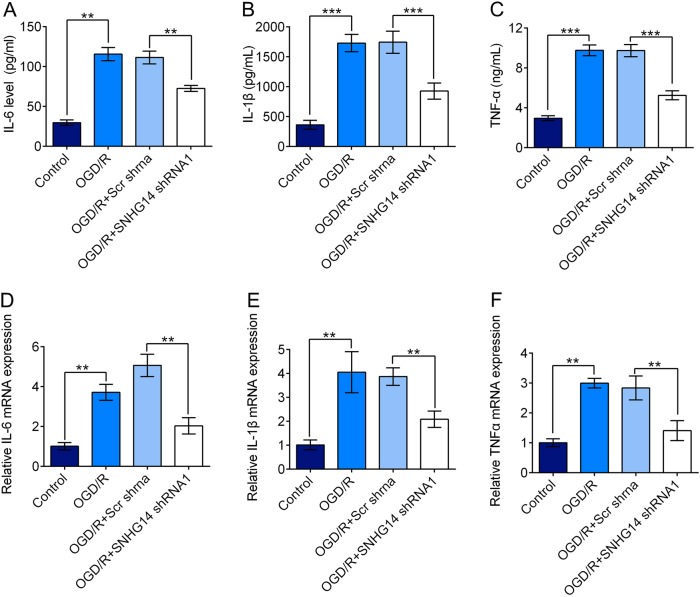
Down-regulated of SNHG14 expression suppressed inflammatory response induced by OGD/R in PC-12 cells. **a**–**c** The effect of transfection of SNHG14 shRNA on the concentration of IL-6, IL-1β, and TNF-α were determined by ELISA assay in OGD/R treated PC-12 cells. **d**–**f** The role of SNHG14 silencing on the mRNA level of IL-6, IL-1β, and TNF-α were detected by qRT-PCR in PC-12 cells under OGD/R treatment. Data were expressed as mean ± SD. **P < 0.01, ***P < 0.001 represents statistically difference

Further, loss of function experiments to evaluate the effect of SNHG14 in response to OGD/R in PC-12 cells were executed. Three shRNAs targeting SNHG14 were examined for their knockdown efficiency. As shown in Fig. 2a, SNHG14 shRNA1, SNHG14 shRNA2, and SNHG14 shRNA3 notably suppressed the expression of SNHG14 in PC-12 cell lines compared with Scr shRNA group. SNHG14 shRNA1 was selected for the further study as its high knockdown efficiency (*P* < 0.05, *P* < 0.01). MTT assay implied that down-regulated expression of SNHG14 enhanced the cell viability in PC-12 cells upon OGD/R treatment (Fig. 2b). The LDH releasing assay showed that the LDH level was dramatically declined after silencing of SNHG14 expression in response OGD/R in PC-12 cells (Fig. 2c). Meanwhile, the caspase-3 activity level and protein level were markedly decreased under OGD/R condition in PC-12 cell lines transfected with SNHG14 shRNA1 compared with Scr shRNA (*P* < 0.01, Fig. 2d, e). Moreover, the typical images showed the calcein-AM labeled live cells (green) and PI labeled dead cells (red), and the percentage of dead cells was remarkably declined treated with SNHG14 shRNA under OGD/R condition (*P* < 0.01, Fig. 2f). In addition, the inflammation related factors were detected by ELISA and qRT-PCR assay. The concentrations or the mRNA expression levels of IL-6, IL-1β, and TNF-α were all reduced by TDNG14 shRNA compared with Scr shRNA in OGD/R injured cells (*P* < 0.01, *P* < 0.001, Fig. 3a–f). Taken together, these data suggested that knockdown of SNHG14 exhibited a neuroprotective role resist I/R injury in vitro.

### SNHG14 reversely modulated miR-136–5p expression by acting as its ceRNA

**Fig. 4:**
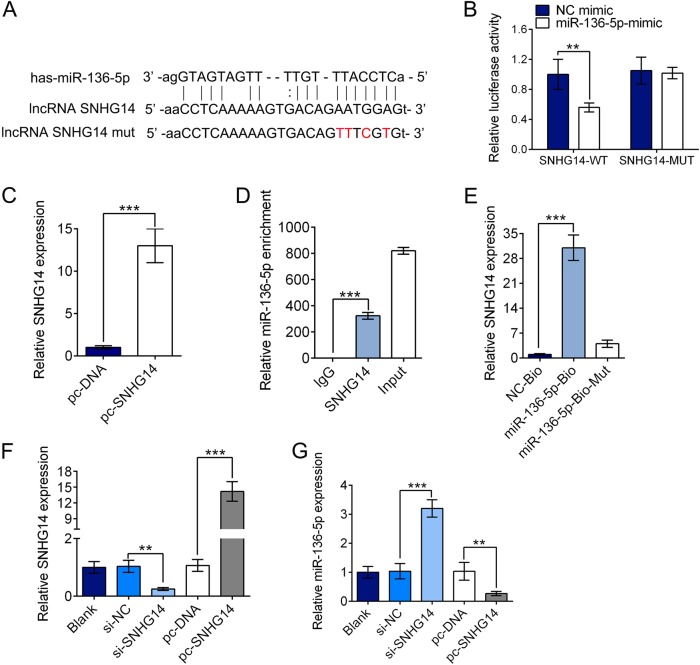
SNHG14 was able to negatively regulate miR-136–5p expression. **a** SNHG14 wide-type (lncRNA SNHG14) and the mutated-type (lncRNA SNHG14 mut) in the miR-136–5p binding sites were shown. **b** Luciferase activity of PC-12 cells co-transfected with miR-136–5p-mimic or NC mimic and luciferase reporters containing SNHG14-WT or SNHG14-MUT transcript were measured by dual-luciferase reporter assays. **c** The expression level of SNHG14 upregulated by transfection with pc-SNHG14 were determined by qRT-PCR. **d** Endogenous miR-136–5p precipitated by AGO2 upon enhanced TDRG1 expression were checked by RIP assay in PC-12 cells. **e** The interaction of SNHG14 and miR-136–5p in PC-12 cells was tested by RNA pull-down assay which precipitated with miR-136–5p-Bio, miR-136–5p-Bio-Mut or NC-Bio, and then examined SNHG14 expression by qRT-PCR. **f** The SNHG14 expressions in PC-12 cells transfected with its siRNA (si-SNHG14) or si-NC, as well as its overexpression vector pc-SNHG14 or pc-DNA were analyzed by qRT-PCR. **g** The effect of downregulated or upregulated SNHG14 by its si-SNHG14 or pc-SNHG14 vector on the expression of miR-136–5p was determined by qRT-PCR. Data were expressed as mean ± SD. **P < 0.01, ***P < 0.001 represents statistically difference

Emerging evidences proved that lncRNAs participate in regulation biological activity via acting as ceRNAs of miRNAs 11. To investigate the mechanism of the function of SNHG14 on ischemia related injury, we screened miRNAs that have complementary base pairing with SNHG14 using online software program starBase (http://starbase.sysu.edu.cn/). miR-136–5p was identified as a potential target of SNHG14 (Fig. 4a), which confirmed by luciferase reporter assays. We generated SNHG14 wild-type (SNHG14-WT) luciferase plasmids containing the potential miR-136–5p binding sites as well as a mutated version of each site (SNHG14-MUT) (Fig. 4a). As shown in Fig. 4b, the luciferase activity in PC-12 cells transfected with SNHG14-WT plasmid was dramatically declined by miR-136–5p-mimic (*P* < 0.01), while no alteration in cells transfected with SNHG14-MUT plasmid (*P* > 0.05). These indicated miR-136–5p is able to bind SNHG14 transcript. Further, RIP and RNA pull down assays were carried out to explore the association between SNHG14 and miR-136–5p. For RIP experiments, the high efficiency for SNHG14 overexpression induced by transfection with pc-SNHG14 was corroborated by qRT-PCR (*P* < 0.001, Fig. 4c). The endogenous miR-136–5p level was specifically enriched in PC-12 cells when highly expressed SNHG14 (*P* < 0.001, Fig. 4d). Further, endogenous SNHG14 was also markedly pulled down by miR-136–5p-Bio but miR-136–5p-Bio-Mut (*P* < 0.001, *P* > 0.05, Fig. 4e). The result revealed the directly interaction between SNHG14 and miR-136–5p. Next, the effect of altered SNHG14 level on miR-136–5p expression was determined by qRT-PCR. The SNHG14 level was decreased by its siRNA while increased by its pc-SNHG14 vector (*P* < 0.01, *P* < 0.001, Fig. 4f). Conversely, the miR-136–5p expression was enhanced by si-SNHG14 but decreased by pc-SNHG14 vector in PC-12 cells (*P* < 0.01, *P* < 0.001, Fig. 4g). In brief, these data demonstrated that SNHG14 negatively regulated the miR-136–5p expression.

### SNHG14 positively modulated ROCK1 expression via miR-136–5p

**Fig. 5:**
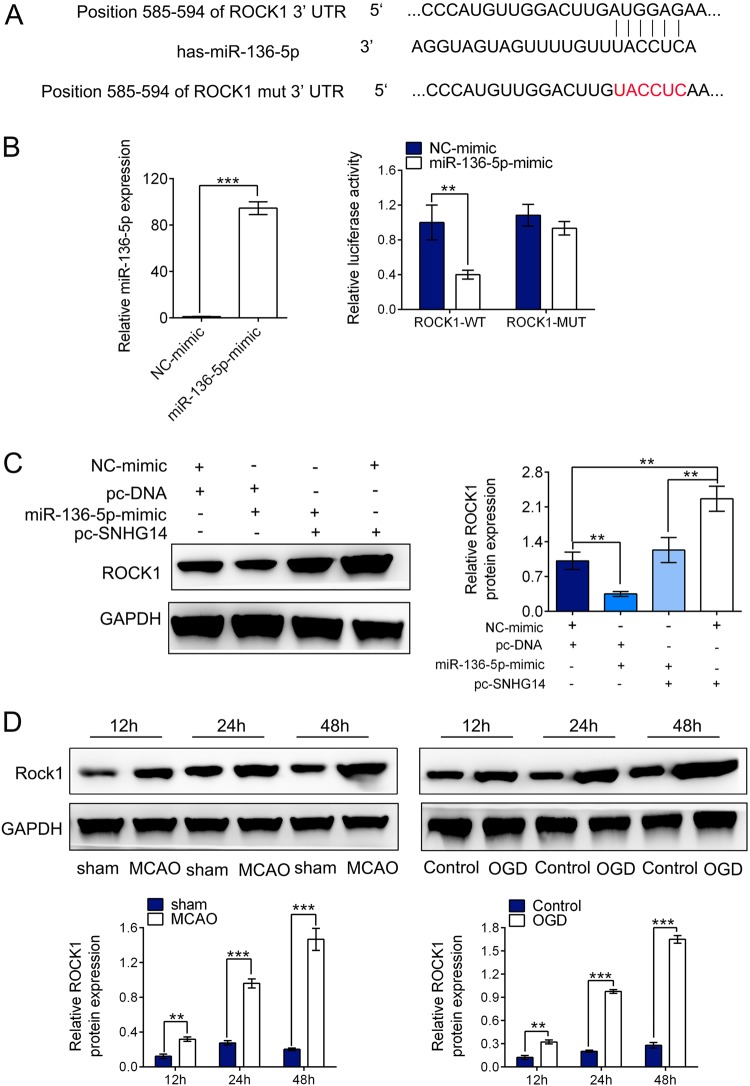
ROCK1 was directly targeted by miR-136–5p and positively regulated by SNHG14. **a** ROCK1 3’UTR wide-type and the mutated-type (ROCK1 3′UTR-MUT) in the miR-136–5p binding sites were shown. **b** The expression level of miR-136–5p was verified by qRT-PCR in PC-12 transfected with miR-136–5p-mimic or NC-mimic. **c** Luciferase activity of PC-12 cells co-transfected with miR-136–5p-mimic or NC-mimic and luciferase reporters containing ROCK1-WT or ROCK1-MUT transcript were analyzed by dual-luciferase reporter assays. **d** The protein level of ROCK1 was detected by western blot in PC-12 cells by co-transfected with SNHG14 overexpression or control plasmid, and miR-136–5p-mimic or NC-mimic. **d** The protein levels of ROCK1 were checked by western blot in rats following MCAO/R at 12, 24, and 48 h or PC-12 cells following OGD/R at 12, 24, and 48 h. Data were expressed as mean ± SD. **P < 0.01, ***P < 0.001 represents statistically difference

Subsequently, bioinformatics analysis using Targetscan (http://www.targetscan.org/) indicated that miR-136–5p bounds to 3′UTR of ROCK1 mRNA (Fig. 5a). We constructed luciferase reporters containing ROCK1 3′UTR wild-type (ROCK1 3′UTR-WT) or a mutant ROCK1 with mutations at the binding sites between ROCK1 mRNA 3′UTR and miR-136–5p (ROCK1 3′UTR-MUT) (Fig. 5a). We firstly validated that miR-136–5p-mimic could elevate the expression of miR-136–5p (Fig. 5b, *P* < 0.001). Dual luciferase reporter assay illustrated that the luciferase activity of PC-12 cells treated with ROCK1 3′UTR-WT was markedly attenuated by miR-136–5p mimic but ROCK 3′UTR-MUT reporter (ROCK1 3’UTR-WT: *P* < 0.01, ROCK1 3′UTR-MUT: *P* > 0.05, Fig. 5b). Next, to explore whether SNHG14 exerted its function on ROCK1 expression via targeting miR-136–5p, after endogenous highly expressed SNHG14 by pc-SNHG14 vector, miR-136–5p expression was further up-regulated by its mimic in PC-12 cells, then the protein level of ROCK1 was determined by western blot. The result showed that up-regulated SNHG14 expression by pc-SNHG14 vector remarkably enhanced the expression of ROCK1, while the increased expression of ROCK1 was blocked by miR-136–5p-mimic (*P* < 0.01, Fig. 5c). Further, ROCK1 expression was examined by western blot in ischemia induced cerebral injury. As shown in Fig. 5d, the protein levels of ROCK1 were heightened in a time dependent manner after reperfusion at 12, 24, and 48 h followed MCAO operation in rats; meanwhile, the expression levels of ROCK1 were gradually elevated at 12, 24, and 48 h after OGD/R treatment in PC-12 cells (*P* < 0.01, *P* < 0.001, Fig. 5d). Altogether, ROCK1 is directly targeted by miR-136–5p but positively regulated by SNHG14.

### SNHG14 modulated ROCK1 expression via miR-136–5p in OGD/R induced inflammatory and neuronal deficit

**Fig. 6:**
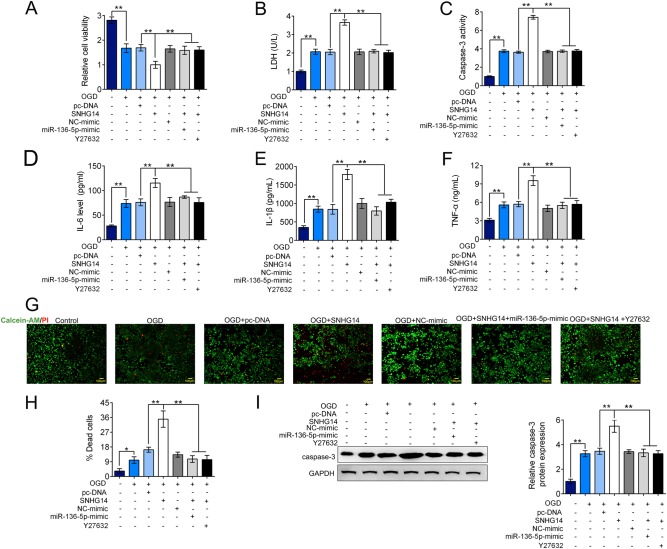
TDRG1 modulated ROCK1 expression via targeting miR-136–5p upon injury and inflammatory response induced by OGD/R. **a**–**h** After treated with OGD/R, PC-12 cells were transfected with SNHG14 overexpression vector pc-SNHG14 or pc-DNA, or co-transfected with pc-SNHG14 and miR-136–5p-mimic, or co-treated with pc-SNHG14 and Y27632 (ROCK1 inhibitor). **a**–**c** The function of enhanced SNHG14 expression, meanwhile with miR-136-5p-mimic or ROCK1 inhibitor on the cell viability, LDH releasing amount or caspase-3 activity was measured by MTT, LDH or caspase-3 activity assay in PC-12 cells under OGD/R treatment, respectively. **d**–**f** The role of increased SNHG14 expression, meanwhile with overexpression of miR-136-5p or ROCK1 inhibitor on the levels of IL-6, IL-1β, and TNF-α were measured by ELISA in PC-12 cells upon OGD/R exposure, respectively. **g**, **h** The effect of upregulated SNHG14 expression accompany with elevated miR-136-5p expression or knockdown of ROCK1 on the number of dead cells were determined by calcein-AM/PI staining in PC-12 cells by OGD/R treatment. **l** The influence of overexpressed SNHG14 accompany with miR-136-5p mimic or knockdown of ROCK1 on the protein level of caspase-3 was checked by western blot in PC-12 cells under OGD/R treatment. Data were expressed as mean ± SD. **P < 0.01 represents statistically difference. Scale bar = 100 μm

To further explore whether SNHG14/miR-136–5p/ROCK1 axis participates in ischemia induced inflammation and injury, we transfected PC-12 cells under OGD/R condition with pc-SNHG14 or pc-DNA alone, or co-transfected with pc-SNHG14 with miR-136–5p-mimic or ROCK inhibitor-Y27632. As shown in Fig. 6a, the cell viability was further decreased by up-regulation of SNHG14 compared with only OGD/R treatment, these reduction was able to be rescued by overexpression of miR-136–5p or inhibition of ROCK1 level (*P* < 0.01, Fig. 6a). However, the apoptotic markers of LDH release and caspase-3 activation were more increased by SNHG14 overexpression compared with only OGD/R treatment but reversely modulated by co-treated with miR-136–5p-mimic or Y27632 (*P* < 0.01, Fig. 6b, c). Conformably, the level of inflammation factors including IL-6, IL-1β, and TNF-α were significantly further elevated by pc-SNHG14 under OGD/R conditions, which increase was down-regulated by miR-136–5p mimic or suppression of ROCK level (*P* < 0.01, Fig. 6d–f). Further, the neuron survival was also verified by calcein-AM/PI dual staining assay and western blot. The percentage of dead cells and apoptotic activator-caspase-3 expression were further enhanced in OGD/R treated PC-12 cells when upregulated SNHG14 expression, however, these elevations were decreased by increasing of miR-136–5p or decreasing of ROCK1 expression (*P* < 0.01, Fig. 6g–i). Collectively, miR-136–5p inhibition or ROCK1 activation is essential for the promotion effect of up-regulated SNHG14 on inflammation and neuron impairment followed OGD insult.

### Knockdown of SNHG14 ameliorated neurological function of MCAO/IR treated rats

**Fig. 7:**
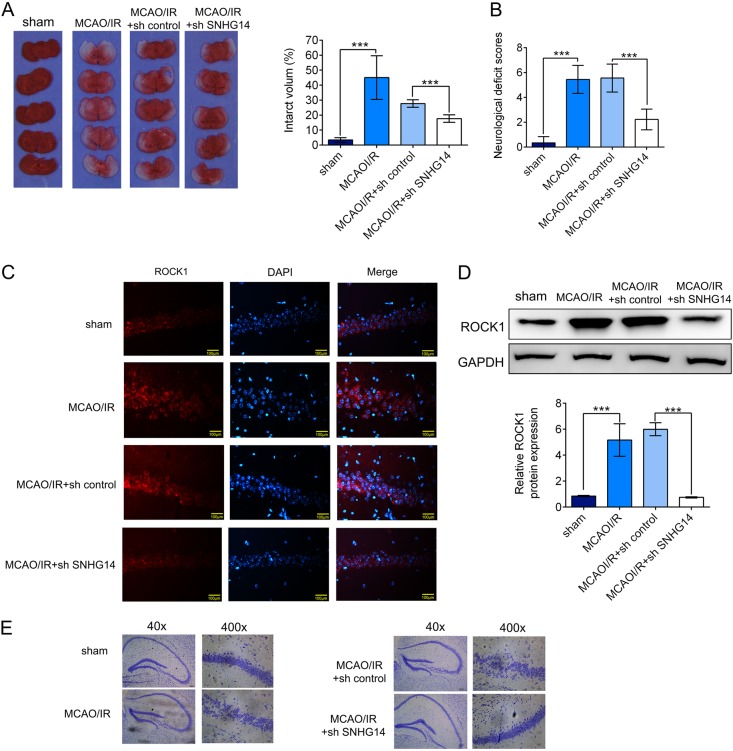
Reduction of SNHG14 level improved neuronal function in MCAO/R treated rats. The animals were injected with lentiviral particle containing sh SNHG14 or sh control into lateral ventricle and 2 weeks later subjected MCAO operation. **a** The effect of SNHG14 silencing on the focal ischemia of brain sections and quantitative analysis of brain infarct volumes was shown by TTC staining in MCAO/R rats. **b** The role of interference of SNHG14 on the neuronal impairment was assessed by neurological deficit scores. **c**, **d** The effect of decreased SNHG14 on the ROCK1 protein level *in vivo* was measured by IF and western blot. **e** Representative images of neuron cells in the hippocampus and cortex were acquired by HE staining in animals injected with TDNG14 lentiviral following MCAO/R. *N* = 6 per group. Data were expressed as mean ± SD. ***P < 0.001 represents statistically difference. Scale bar = 100 μm

Further, MCAO/IR rats were intraventricular injected by SNHG14 knockdown lentiviral to assess the effect of SNHG14 silencing on neurological function in vivo. As shown in Fig. 7a, the infarct volume and brain damage were dramatically decreased by sh SNHG14 compared with sh control group in MCAO/R treated rats (*P* < 0.001). Moreover, the neurological behavior was also ameliorated by interference of SNHG14 compared with sh control in MCAO/R which evaluating by the neurological deficit scores (*P* < 0.001, Fig. 7b). Next, IF and western blot were performed to check the ROCK1 level in vivo. Compared with sh control group, the protein level of ROCK1 was attenuated in sh SNHG14 treated group in MCAO/R rats (*P* < 0.001, Fig. 7c, d). For further grouped MCAO/R treated animals, knockdown of SNHG14 relieved the effect of I/R on cortex and hippocampus tissues compared with sh control group (Fig. 7e). These data suggested knockdown of SNHG14 promoted neurological function via decreasing ROCK1 expression in vivo.

## Discussion

The identification of effective treatment tool is pivotal in ischemia stroke as it causes high rates of death and disability worldwide. Recently, non-coding RNAs are spring up to be as novel therapeutic targets in ischemia stroke [22, 23]. In present study, we demonstrated that lncRNA SNHG14 expression was upregulated in MCAO/R treated brain tissues and OGD/R treated PC-12 cells. Interference of SNHG14 by its shRNA promoted neuron survival and inhibited inflammatory response to OGD/R insult. SNHG14 positively regulated the expression of ROCK1 via acting as a sponge of miR-136–5p in vitro and in vivo. SNHG14 enhanced neuronal deficit and inflammation through upregulation of ROCK expression while down-regulation of miR-136–5p in OGD/R injury. Deletion of SNHG14 expression improved neurological function in vivo. Finally, we demonstrated that SNHG14 participated in regulation of inflammation induced by I/R damage via regulation of miR-136–5p/ROCK1 axis and could be a new strategy for the treatment cerebral damage induced by ischemia.

Several studies revealed that SNHG14 was related with central neuronal system diseases [10, 24]. As its locus overlaps with UBE3A gene and promoter, thus SNHG14 prevents UBE3A expression and leads to neurogenetic disorders, such as Angelman syndrome [8, 10]. In addition, SNHG14 was reported as inhibitor in malignant glioma by suppressing cell growth, invasion, and promoting apoptosis in glioma cell lines via targeting miR-92a-3p [24]. Further, SNHG14 was involve in regulation of microglia activation in cerebral infarction [9]. Consistent with our study, SNHG14 was also found to be highly expressed in MCAO mice and OGD/R BV-2 cells [9]. And SNHG14 knockdown suppressed microglia cells activation and inflammatory response through enhancing PLA2G4A but reducing miR-145–5p level in OGD treated cells 9. In our study, we also found interference of SNHG14 improve ischemia induced injury and inflammation in vitro and vivo. We strengthened the new evidence of SNHG14 in cerebral stoke, which further confirmed by in vivo experiments. It maybe more interesting to measure whether overexpression of TNRG14 could promote the injury or inflammation in normal animal.

To act as the ceRNA of miRNA is a major regulatory mechanism for lncRNA [25]. Currently, miR-136–5p was found as a target miRNA of SNHG14. miR-136 exerted protective functions on neuron cell against spinal cord ischemia injury via targeting metalloproteinases-3 [26]. Consistently, miR-136 could resist cisplatin resistance and promote the response to cisplatin treatment via suppressing E2F transcription factor 1 [27]. Our present study also revealed that miR-136–5p blocked ischemia induced injury and up-regulation of miR-136–5p attenuated the aggravated damage induced by SNHG14 overexpression in OGD/R induced cells. In addition, miR-136 also participated in modulation of inflammatory response in neuronal disease [28]. He et al. showed that miR-136–5p enhanced astrocytes producing inflammatory factors and chemokines by increasing the expression of p-NF-κB and decreasing A20 after spinal cord injury [28]. However, we found that miR-136–5p blocked the inflammatory response induced by up-regulation of SNHG14. Maybe, the one miRNA plays different roles in various diseases.

Previous researches have reported that ROCK1 related RohA/Roh-kinase contributes to regulation of ischemia related diseases [29]. Most studies focus on the important roles of ROCK1 in pathogenesis of cardiac system ischemia disease [30], only few studied its function in cerebral ischemia. Geng et al. demonstrated that Yes-associated protein protect N2a cell from cerebral I/R insult via blocking ROCK1/F-actin signaling pathway [20]. Similarly, we illustrated that ROCK1 is up-regulated in ischemia induced injury in vivo and in vitro, and preventing its expression ameliorated SNHGA14 overexpression aggravated OGD/R induced deficit and inflammation.

In conclusion, we illustrated that lncRNA SNHG14 was highly expressed in vivo and in vitro, and promoted neuronal damage and inflammation induced by ischemia injury. Mechanically, SNHG14 silencing improved neurological function and prevented inflammation dependent on miR-136–5p overexpression and in turn decreased ROCK1 level. SNHG14 is injurious to neuron survival induced by ischemia insult, which providing a novel therapeutic strategy and drug target for clinically treatment ischemia stroke.

## Electronic Information

Sup Legend

Sup Fig
