# Supplementary material for: LncRNA SNHG14 promotes inflammatory response induced by cerebral ischemia/reperfusion injury through regulating miR-136-5p /ROCK1
Source: Cancer Gene Ther. 2018 Dec 14;26(7):234–47. doi: 10.1038/s41417-018-0067-5 (PMC6760557; doi:10.1038/s41417-018-0067-5)
Supplement: Supplementary file 1 — Sup Legend [file 41417_2018_67_MOESM1_ESM.docx]

**Sup Figure:** The mycoplasma test of PC-12 cells were performed by staining with Hoechst 33258. Scale bar = 50 μM.
