# Supplementary figures and images for: LncRNA SNHG14 promotes inflammatory response induced by cerebral ischemia/reperfusion injury through regulating miR-136-5p /ROCK1
Source: Cancer Gene Ther. 2018 Dec 14;26(7):234–47. doi: 10.1038/s41417-018-0067-5 (PMC6760557; doi:10.1038/s41417-018-0067-5)

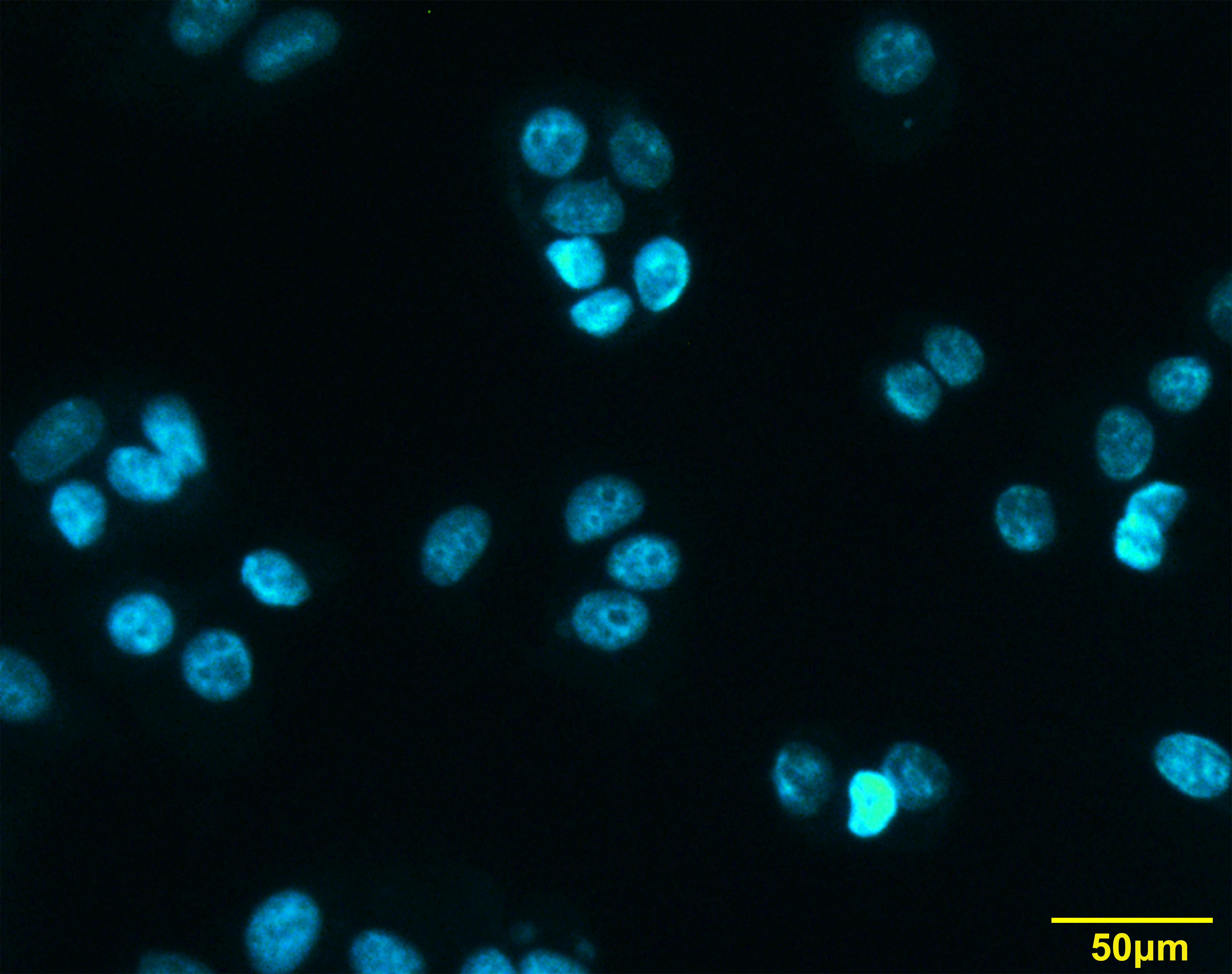

Supplement: Supplementary file 2 — Sup Fig [file 41417_2018_67_MOESM2_ESM.tif]
